# High GP73 Expression Correlates with Poor Response to Neoadjuvant Chemotherapy and Survival in Gastric Cancer: A Tissue Microarray Study

**DOI:** 10.3389/pore.2021.603838

**Published:** 2021-03-30

**Authors:** Jian Guo Shen, Jun Shen, Rong Yue Teng, Lin Bo Wang, Wen He Zhao, Qin Chuan Wang

**Affiliations:** Department of Surgical Oncology, Sir Run Run Shaw Hospital, Zhejiang University School of Medicine, Hangzhou, China

**Keywords:** GP73, gastric cancer, prognosis, neoadjuvant chemotherapy, pathological response

## Abstract

Golgi protein 73 (GP73) is a type II Golgi transmembrane protein which is overexpressed in several cancers, however, its role in gastric cancer is still unclear. The aim of this study is to investigate if high GP73 expression is associated with pathological tumor response to neoadjuvant chemotherapy and prognosis for patients with gastric cancer. A total of 348 patients with gastric cancer, who had undergone surgery between 1999 and 2011 were retrospectively reviewed, GP73 expression was examined in tumor tissues using tissue microarray and the correlations between its expression and pathological response to neoadjuvant chemotherapy as well as patients prognosis were analyzed. We found that GP73 expression was not associated with clinicopathologic features including tumor size, differentiation and TNM stage. High expression of GP73 was associated with less pathological tumor response to neoadjuvant chemotherapy and poor survival in gastric cancer, multivariate analysis showed GP73 expression was an independent predictive factor for pathological response to neoadjuvant chemotherapy and for prognosis in patients with gastric cancer. Our results suggest that GP73 expression correlates with the effect of neoadjuvant chemotherapy and is a promising biomarker to identify patients with poor prognosis.

## Introduction

Gastric cancer (GC) is now the fourth most common cancer and is the second most common cause of death from cancer in the world. It is estimated that two-thirds of gastric cancer cases occur in developing countries and 42% in China alone [1, 2]. Surgery remains the first choice of treatment for GC. However, the 5-years survival rate for patients with all stages stays ∼20% [3]. Thus, it is essential to develop effective prognostic factors that may predict patient survival in gastric cancer.

Preoperative chemotherapy is now widely used in patients with locally advanced gastric cancer, since it can improve complete surgical resection so as to improve survival [4]. However, the effect of neoadjuvant chemotherapy stays at 50%, in unresponsive cases, it may have potential to delay surgical treatment [5]. Therefore, tailored therapy may be conducted if factors predicting the response of neoadjuvant chemotherapy can be preoperatively identified in gastric cancer.

GP73, also known as GOLM1 and GOLPH2, is a type II Golgi protein which is normally located within the cis-Golgi complex [6]. Most studies showed that GP73 was overexpressed and was correlated with tumor progression and poor survival in patients with several types of cancer including hepatocellular carcinoma, colon cancer [7, 8]. The association between GP73 and gastric cancer remains controversial, GP73 was reported to be correlated with tumor differentiation in gastric cancer [9], however, another study found a correlation between GP73 and patients survival [10].

In this study, we evaluated GP73 expression by immunohistochemistry (IHC) on a tissue microarray containing 348 gastric cancer tumor tissues, and tried to investigate the expression of GP73 and its relationship with pathological response in patients with neoadjuvant chemotherapy as well as prognosis in patients with gastric cancer.

## Materials and Methods

### Ethic Statement

The protocol of this study was reviewed and approved by the institutional review board (IRB) of Zhejiang University Affiliated Sir Run Shaw Hospital (SRRSH) (Approval code: 2016-0628-3). Written informed consent was obtained from all the patients enrolled in this study.

### Patients and Tissue Samples

A total of 348 patients with gastric cancer who underwent surgical treatment in the department of surgical oncology, Sir Run Shaw hospital between 1996 and 2011 were enrolled into the study. Patient demographics and clinicopathologic characteristics, including tumor size, location, depth of invasion, tumor differentiation, node status and TNM stage were documented. Patients clinically staged as T2 and above or having lymph node metastasis were recommended to have neoadjuvant chemotherapy, and the effect of chemotherapy was assessed based on the RECIST criteria. Surgical treatment was generally performed according to the rules of the Japanese Research Society for Gastric Cancer. After surgery, tumor specimens were sent to the Pathology and the pathologic stage was determined according to the rules of the sixth edition of UICC and the stage grouping of the UICC/AJCC. Pathological response was evaluated among the patients with neoadjuvant chemotherapy by using tumor regression grade (TRG), which was proposed by Mandard’s et al. [11]. Briefly, cancer with complete regression was graded as TRG4, isolated cell nests as TRG 3, more residual cancer cells but fibrosis still predominates as TRG 2 and residual cancer outgrowing fibrosis or absence of regressive changes as TRG 1. Generally, TRG 3 and 4 were classified as tumor regression, which was referenced in previous studies. The patients were followed up until death or until the date of last follow-up of Feb 28, 2015. The median follow up time was 22.0 months (range, 1–182 months).

### TMA Preparation and IHC

GP73 expression was evaluated in 348 gastric cancer tissues by IHC in TMA. The construction of TMA was previously reported [12]. Briefly, all the slides were reviewed by the pathologist from SRRSH. Then, the tissue blocks were retrieved and labeled for biopsy. All the selected samples were biopsied and reassembled into a paraffin multiple tissue carrier as a set of TMA. Each case had three cores in the TMA. The 5-μm-thick slices of TMA were prepared and stored at 4°C until use. GP73 was IHC stained in the TMA, The staining condition was validated using negative and positive tissue controls for GP73 antibodies. IHC was performed as we previously described [12]. Briefly, following deparaffinization, 3% H_2_O_2_ (hydrogen peroxide) was used to block the endogenous peroxidase activity. The array slides were later incubated with normal goat serum, then the primary (GP73 antibody, 1:1000, Hotgen, Beijing) and secondary (Rabbit) antibodies were applied accordingly. DAB (3,39-diaminobenzidine; 0.05 g DAB and 100 ml of 30% H2O2 in 100 ml of PBS) was used for specific staining. Each slide was then counterstained with hematoxylin (DAKO). Hepatocyte and PBS was used as positive control and negative control, respectively.

### Interpretation of IHC GP73 Expression on TMA

The IHC staining of cytoplasmic GP73 were evaluated by two pathologists independently, based on the intensity and the proportion of positively stained cancer cells. The proportion of positively stained cancer cells was scored as follows: 0 for no positive tumor cells, 1 for <10% positive tumor cells, 2 for 10–35% positive tumor cells, 3 for 35–70% positive tumor cells and 4 for >70% positive tumor cells. The intensity was scored as: 0 (no staining), 1 (weak staining), 2 (moderate staining) and 3 (strong staining). Scores for intensity and proportion of positive cells were multiplied as final histoscores of the samples. Scores ≤3 was regarded as tumors with low or no GP73 expression and scores ≥4 as high GP73 expression.

### Statistical Analysis

All demographic data, clinicopathologic variables and IHC results were coded and entered into a gastric cancer database. Double data entry and logic checks were used for error reduction. All statistical analyses in the study were performed by SPSS 22.0 (SPSS, Chicago, IL, United States). The Chi-square test and Fisher’s exact test were performed to assess the correlations between GP73 expression level and clinical characteristics. Kaplan–Meier method was used to calculate the survival probabilities and log rank test was used to compare survival curves. Independent factors influencing the survival were determined by multivariate analysis using Cox regression model. Factors predicting neoadjuvant pathological response were determined by means of logistic regression analysis. A *p* value of less than 0.05 was considered statistically significant.

## Results

### Correlation Between GP73 Expression and Clinicopathologic Parameters

**FIGURE 1:**
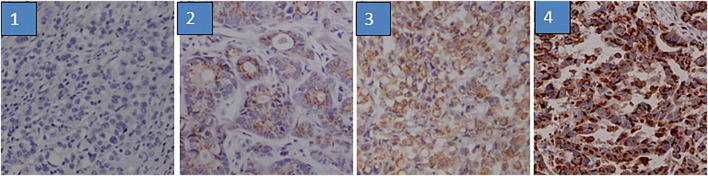
Immunohistochemistry staining of GP73 according to its expression. The IHC staining of GP73 in TMA was evaluated based on staining intensity and proportion of positively stained tumor cells. 1. negative staining (score 0); 2, weak staining (score 1); 3, medium staining (score 2); 4, strong staining (score 3).

GP73 expression was determined by IHC in 348 gastric cancer tissues on the TMA. GP73 was mainly localized in the cytoplasm of the cancer cells (Figure 1). A total of 152/348 gastric cancer tissues (43.7%) exhibited high GP73 expression, while 196/348 (56.3%) tissues were shown to have low or no GP73 expression. The correlations between GP73 expression and clinical characteristics are shown in Table 1. Patients with larger tumor size were more likely to have high GP73 expression than those with small tumor size (*p* = 0.014), however, other clinicopathologic parameters including age, tumor location, differentiation, peritoneal metastasis and TNM stage were not associated with the GP73 expression level.

**TABLE 1:** Correlation between GP73 expression and clinicopathologic factors of 348 gastric cancer patients.

Characteristics	GP73		*p*-value
	Low or no. of cases (%)	High no. of cases (%)	
Age (Mean ± SD, years)	58.1 ± 11.2	60.3 ± 12.9	0.087
Gender			0.368
Female	58 (54.2)	49 (45.8)	
Male	133 (56.8)	101 (43.2)	
Unknown	5 (71.4)	2 (28.6)	
Tumor location			0.335
Proximal	33 (53.2)	29 (46.8)	
Middle	48 (64.0)	27 (36.0)	
Low body	99 (52.7)	89 (47.3)	
Whole	6 (66.7)	3 (33.3)	
Unknown	10 (71.4)	4 (28.6)	
Tumor size (Mean ± SD, cm)	5.1 ± 2.7	5.8 ± 2.5	0.014
Differentiation			0.898
Well or moderate	56 (57.1)	42 (42.9)	
Poor or no	94 (55.6)	75 (44.4)	
Unknown	46 (55.4)	35 (44.6)	
T classification			0.109
T1+T2	58 (63.7)	33 (36.3)	
T3+T4	132 (53.9)	113 (46.1)	
Unknown	6 (50)	6 (50)	
LN metastasis			0.457
No	54 (60)	36 (40)	
Yes	132 (55.2)	107 (44.8)	
Unknown	10 (52.6)	9 (47.4)	
Peritoneal metastasis			0.657
No	192 (56.3)	149 (43.7)	
Yes	2 (40.0)	3 (60.0)	
Unknown	2 (100)	0 (0)	
TNM stage			0.503
I + II	84 (59.2)	58 (40.8)	
III + IV	106 (54.9)	87 (45.1)	
Unknown	6 (46.1)	7 (53.9)	
Neural invasion			0.516
No	171 (56.1)	134 (43.9)	
Yes	24 (57.1)	18 (42.9)	
Unknown	1 (100)	0 (0)	
Vascular invasion			0.208
No	185 (56.9)	140 (43.1)	
Yes	9 (45.0)	11 (55.0)	
Unknown	2 (66.7)	1 (33.3)	
Carcinomatous nodule			0.273
No	172 (57.5)	127 (42.5)	
Yes	23 (47.9)	25 (52.1)	
Unknown	1 (100)	0 (0)	
Type of surgery			0.075
Radical	157 (58.8)	110 (41.2)	
Palliative	37 (48.7)	39 (51.3)	
Unknown	2 (40.0)	3 (60.0)	

### Correlation of GP73 Expression With Patients Overall Survival

**FIGURE 2:**
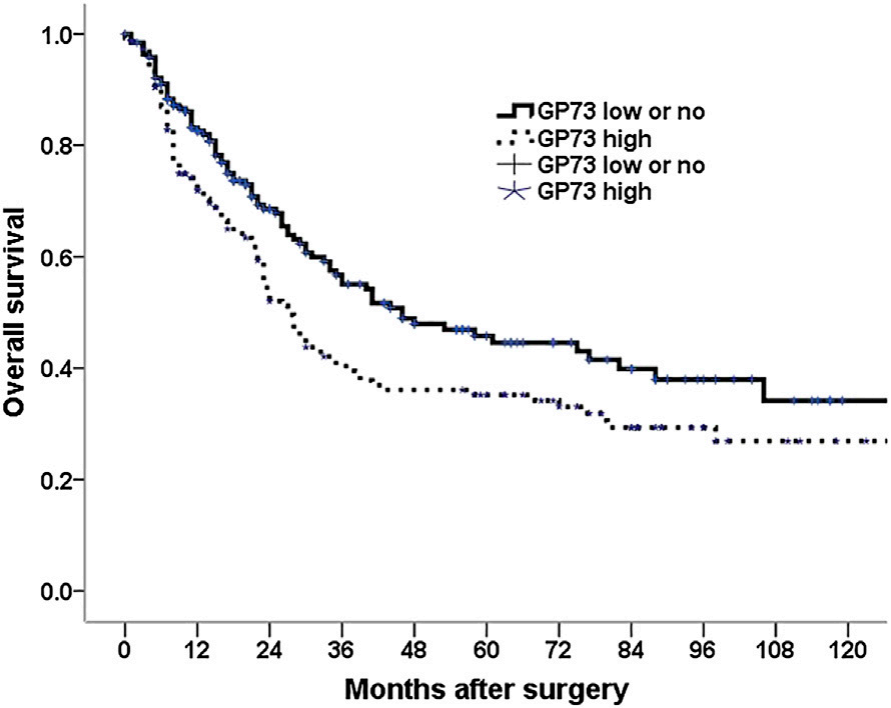
Kaplan–Meier survival analysis for 348 gastric cancer patients with a low and no vs. a high GP73 expression tumors.

Among the 348 patients, 179 patients (51.4%) died during the follow up period. Univariate analysis was conducted to investigate the relationship between the clinicopathologic characteristics and patients overall survival, we found that patient age, tumor location, tumor size, differentiation, neural invasion, carcinomatous nodule, tumor depth of invasion, lymph node metastasis, TNM stage, type of surgery as well as GP73 expression were associated with patients survival. Patients with high GP73 expression observed a shorter median overall survival than those with low or no GP73 expression (Figure 2). However, patient gender, peritoneal metastasis as well as vascular invasion were not variables influencing the patients’ prognosis (Table 2).

**TABLE 2:** Univariate and multivariate analysis of gastric cancer with survival.

	Univariate analysis		Multivariate analysis	
	Hr (95% CI)	*p* Value	Hr (95% CI)	*p* Value
Age (<60 vs. ≥60 years)	1.105 (1.000–1.029)	0.044	1.021 (1.001–1.042)	0.041
Gender (female vs male)	0.929 (0.587–1.470)	0.753		
Tumor location (P/W vs. M/L)	0.545 (0.339–0.876)	0.012	0.912 (0.696–1.194)	0.502
Tumor size (<4 vs. ≥4 cm)	1.144 (1.069–1.224)	0.000	1.172 (0.643–2.136)	0.604
Differentiation (well vs. Poor)	1.591 (1.014–2.497)	0.044	0.977 (0.784–1.218)	0.837
Depth of invasion (T1,2 vs. T3,4)	5.489 (2.745–10.977)	0.000	2.060 (0.926–4.585)	0.077
LN metastasis (no vs. Yes)	2.600 (1.462–4.623)	0.001	0.745 (0.312–1.779)	0.507
Peritoneal metastasis (no vs. Yes)	1.624 (0.401–6.575)	0.497		
TNM stage (I, II vs. III, IV)	1.934 (1.519–2.460)	0.000	1.843 (1.220–2.785)	0.004
Neural invasion (no vs. Yes)	2.794 (1.701–4.588)	0.000	2.099 (1.204–3.659)	0.009
Vascular invasion (no vs. Yes)	1.202 (0.523–2.763)	0.664		
Carcinomatous nodule (no vs. Yes)	2.612 (1.252–5.447)	0.010	2.142 (0.996–4.607)	0.051
Type of surgery (radical vs. palliative)	4.920 (3.415–7.086)	0.000	2.584 (1.557–4.288)	0.000
GP73 expression (low or no vs. high)	1.681 (1.095–2.579)	0.018	1.636 (1.043–2.567)	0.032

P/W: proximal/whole body; M/L: middle/low body.

Multivariate analysis using the Cox proportional hazard model was employed to identify the prognostic factors in gastric cancer patients, all variables that were significant in univariate analysis were entered into the model. Our results showed that high GP73 expression was one of the independent prognostic factors for survival in gastric cancer patients (Table 2).

### Efficacy of Neoadjuvant Chemotherapy in Low or No- and High GP73 Expressing Patients

Forty-three out of 348 patients (12.4%) with locally advanced gastric cancer received neoadjuvant chemotherapy before surgery, among them, 74.4% of patients treated with oxaplatin-based regimens and the others (25.6%) with docetaxel-based regimens, and 55.8% patients completed more than three cycles of neoadjuvant chemotherapy before surgery. Nine patients (20.9%) showed high GP73 expression and 34 patients showed low or no GP73 expression. Pathologic tumor response after neoadjuvant chemotherapy was routinely assessed by the pathologist by using TRG. TRG1 was observed in 17 patients (39.5%), TRG2 in 16 patients (37.2%), TRG3 in 8 patients (18.6%) and TRG4 in 2 patients (4.7%), respectively. Ten patients (23.3%) showed tumor regression (TRG 3 or 4) as defined in the present study, and 2 patients showed complete regression.

The relationship between neoadjuvant pathological response and clinicopathologic parameters as well as treatment variables including age, gender, tumor staging, tumor location, differentiation, tumor size, peritoneal metastasis, neural invasion, vascular invasion and chemotherapy regimens were evaluated using univariate analysis, univariate predictors of tumor regression were found to be tumor size and GP73 expression. Patients with larger tumor size showed less tumor regression as compared with those with smaller tumor size (*p* = 0.027). Patients with high GP73 expression represented less tumor regression as compared to those with low or no GP73 expression (*p* = 0.023) (Table 3). Multivariate analysis using Logistic regression model identified GP73 as one of the independent predictive factors for pathological response (*p* = 0.045, Odds Ratio, 10.647, 95% CI for odds ratio, 1.050–107.954) (Table 4).

**TABLE 3:** Correlation between GP73 expression and tumor regression grade of 43 gastric cancer patients with neoadjuvant chemotherapy.

	GP73 expression		*p*-value
	Low or no. of cases (%)	High no. of cases (%)	
Tumor regression grade			0.023
1	10 (29.4)	7 (77.8)	
2	14 (41.2)	2 (22.2)	
3 and 4	10 (29.4)	0 (0)	

**TABLE 4:** Multivariate logistic analysis to identify predictors of tumor regression in patients with neoadjuvant chemotherapy.

Variables	Odds ratio	95% CI	*p* Value
GP73 expression (high vs. low or no)	10.647	1.050–107.954	0.045
Tumor size (cm) (continuous)	1.494	1.060–2.108	0.022

## Discussion

In the present study, we found that high GP73 expression was associated with poor outcome in patients with gastric cancer and was an independent prognostic factor for survival. To the best of our knowledge, this is the first study to evaluate the correlation between GP73 expression and pathological response to chemotherapy in gastric cancer. Patients with high GP73 expression were more likely to obtain less pathological tumor regression as compared to patients with low or no GP73 expression, these results indicate that GP73 correlates with the effect of neoadjuvant chemotherapy in advanced gastric cancer.

GP73 was originally cloned from a library derived from the liver tissue of a patient with adult giant-cell hepatitis [13]. The role of GP73 are mainly studied in hepatocellular carcinoma, high-expression of GP73 was associated with tumor size, differentiation, grade and survival, which indicate that GP73 is a valuable marker using as an independent diagnostic tool for hepatocellular carcinoma [14–18]. However, knowledge on the function of GP73 is limited in gastric cancer.

The relationship between GP73 expression and gastric cancer progression is still controversial. A study from Chen et al. assessed GP73 protein expression by immunohistochemistry in both tumor and non-tumorous gastric mucosal tissue, they found GP73 was down-regulated in gastric cancer, and its expression in gastric cancer was associated with tumor differentiation [9]. Liu et al’s study revealed a significant correlation between GP73 expression and clinical stage, lymph node metastasis and venous invasion, thus the study made a conclusion that GP73 expression may be associated with tumor progression [10], However, in the present study, no significant correlation was found between GP73 expression and clinical variables including age, differentiation and TNM stage, this discrepancy may be explained as the following: firstly, a comparatively more gastric cancer samples were enrolled in the present study, the difference of patient clinical characteristics as a selection bias may influence the analysis. Secondly, we assessed GP73 expression by IHC on TMA, and it may, to some extent, reduce the experimental bias as compared to IHC on separate sections. Finally, the difference of interpretation of GP73 IHC results may also affect the results we achieved.

The prognostic effect of GP73 expression was reported in some cancers. A study from Jiang et al showed that patients with high GP73 expression achieved poorer outcome than those with low or no GP73 expression in hepatocellular carcinoma (HCC) [17], however, Sun et al’s reports showed no survival difference between both groups in terms of GP73 expression [18]. In the present study, we observed that patients with high GP73 expression had a significantly lower overall survival rate than those with low or no GP73 expression, and multivariate analysis revealed GP73 expression was an independent predictor for survival. These results were in accordance with a study of Liu et al, who analyzed the GP73 expression by IHC in 385 gastric cancer patients, and found that GP73 was a useful prognostic variable of overall survival in gastric cancer patients [10]. Our results, with previous reports, may indicate that GP73 may have the potential to be a new target in the treatment of gastric cancer.

A recent randomized phase III trial demonstrated a survival benefit for gastric cancer patients with perioperative chemotherapy when compared with surgery alone [4], and tumor regression grade was mainly utilized as a reasonable method for predicting pathologic response of cytotoxic agents [19], thus, it is essential to identify clinical markers predicting the pathologic tumor response before treatment. In the present study, we found that patients with high GP73 expression were more likely to obtain less tumor response than those with low or no GP73 expression, and further multivariate analysis found that GP73 expression served as one of the important predictors of pathologic tumor regression. These results were based on assessment of GP73 expression in post-treatment surgical samples since the biopsy specimens were not available because, for most of these patients, pretreatment endoscopy was not performed in our institution. We have started to validate these findings by determining GP73 expression in pretreatment biopsy specimens.

The mechanism of the correlation between GP73 expression and chemotherapy response in cancer is unclear. Ye et al reported that GP73 may significantly change cell proliferation and apoptosis so as to influence the oxaplatin resistance in hepatic carcinoma cells [20]. Zhou et al’s study found that GP73 induced cisplatin resistance in HT29 colon cancer cells was related to the activation of the mitogen-activated protein kinase/ERK and Wnt/β-catenin signaling pathways [21]. However, the interaction between GP73 and chemoresistance in gastric cancer was rarely reported, and thus needs to be further studied.

Based on our data, there were some weaknesses in the study. Firstly, as we know now, tumor histological type was associated with the response rate to neoadjuvant chemotherapy in gastric cancer, however, data of histological type was not available in this study and the distribution of GP73 expression by histological type was unclear. Secondly, most of patients enrolled in the study were treated before 2010, thus, patients staging were determined by the sixth edition of the TNM system, it is unclear whether the influence of GP73 expression on prognosis will be changed if the latest edition of TNM staging is used in this study. Finally, our results were based on a small number of patients’ analysis with a short median follow-up time and need to be validated in large-scale studies.

In conclusion, our results suggest that GP73 correlates with the effect of neoadjuvant chemotherapy in gastric cancer and may serve as a promising biomarker to identify patients with poor prognosis, however, its potential role in the management of patients with gastric cancer needs to be further studied.

## Data Availability

The raw data supporting the conclusions of this article will be made available by the authors, without undue reservation.

## Abbreviations

GP73, Golgi protein 73; TMA, tissue microarray; GC, Gastric cancer; IHC, immunohistochemistry; TRG, tumor regression grade; HCC, hepatocellular carcinoma
